# Unexpected Stability of a Prodrug to Enzymatic Hydrolysis within a Hydrated HPMC Matrix Tablet

**DOI:** 10.3390/pharmaceutics14102222

**Published:** 2022-10-18

**Authors:** Sarah Hanley, Jonathan Brown, Peter Timmins, Catrin Davies, Andrew Dennis

**Affiliations:** Drug Product Development, Bristol Myers Squibb, Reeds Lane, Moreton, Merseyside CH46 1QW, UK

**Keywords:** hydrophilic matrix tablet, alkaline phosphatase, stability, prodrug

## Abstract

The uptake of alkaline phosphate present in dissolution medium into a hydrating hydroxypropyl methylcellulose matrix tablet and that its activity was retained therein was demonstrated. This presents a risk to the stability of prodrugs that are substrates of this enzyme such as phosphonooxymethyl derivative prodrugs. It was found that fostemsavir, a phosphonooxymethyl derivative prodrug being developed for the treatment of HIV-1 infection, was unexpectedly resistant to hydrolysis within a hydrated HPMC matrix when subjected to drug release testing in media containing alkaline phosphatase. Studies indicated that this was not due to microenvironmental pH effects, osmolality effects or effective phosphate concentration effects associated with the presence of the prodrug. That the prodrug and not its parent could affect enzyme activity in a concentration dependent manner, and that another phosphate ester prodrug fosphenytoin did not inhibit alkaline phosphatase activity within a hydrated HPMC matrix suggested that the unexpected stability of the HIV-1 therapy prodrug may be associated with the ability of the phosphate group-containing compound itself to inhibit the enzyme at the concentrations it exists at in the hydrated dosage form and so enables the development of the compound in this type of dosage form.

## 1. Introduction

Although there have been significant advances in the development of therapies to treat patients infected with human immunodeficiency virus type 1 (HIV-1) over the last three decades, leading to reduction in its incidence and longer life expectancy of infected individuals, the introduction of new antiretroviral agents remains essential, to overcome resistance development to existing classes of drugs and to manage safety and tolerability challenges with currently available therapies [1,2].

**Figure 1:**
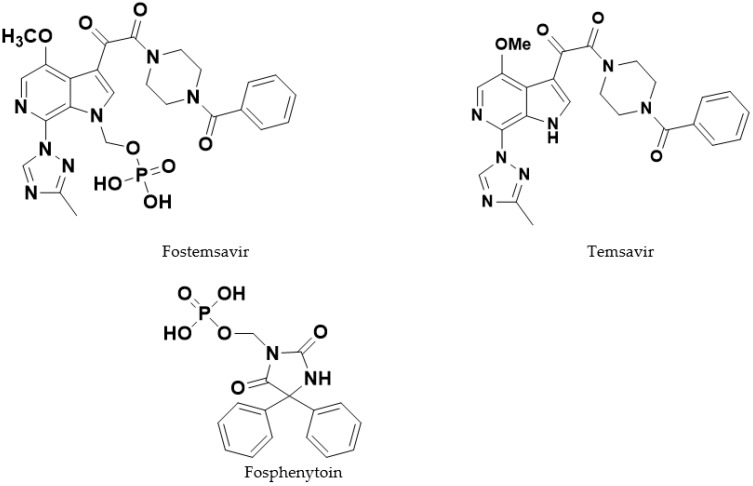
Structures of prodrug fostemsavir, its parent compound temsavir and fosphenytoin.

Recently, a novel class of HIV-1 attachment inhibitors have been described that prevent the initial interaction between virus and host cell by binding to the gp120 protein on the viral envelope and thus interfere with the attachment of the virus to the CD4 receptor on CD4+ T-cells [3]. As is the case with many recently developed agents for the treatment of HIV infection [4] the therapeutic utility of these agents was challenged by their poor solubility and pharmacokinetic properties, requiring enabling approaches to be employed to allow the investigation of these agents in the clinic [5]. Fostemsavir (Figure 1) is an HIV-1 attachment inhibitor in this novel class which was discovered by Bristol Myers Squibb [6], acquired by ViiV healthcare in 2016 [7] and is under development for the treatment of HIV/AIDS [8]. It gained initial regulatory approval in 2020 [9].

The molecule is a phosphonooxymethyl prodrug (referred to subsequently in this article as a “phosphate ester prodrug”) of the pharmacologically active parent compound temsavir (Figure 1). Prodrugs have been previously used as successful strategies in HIV therapy, as they can overcome solubility-limited absorption of the parent drug [10]. Fostemsavir, as its tromethamine salt, is freely soluble (>250 mg/mL in water, compared with the parent temsavir which is practically insoluble, 0.022 mg/mL in water at pH 5.7) and hence was selected for development.

Phosphate ester prodrugs have been already demonstrated as an approach to improve oral bioavailability of a number of poorly soluble compounds [11,12]. The addition of an ionizable phosphate moiety increases the solubility of the compounds at physiological pH, enabling rapid dissolution in the GI contents. These prodrugs are designed to convert back to the active parent compound at the brush border surface of the intestinal epithelial surface where alkaline phosphatase is abundantly present [13]. When using a prodrug strategy, the resistance to precipitation of the parent compound is very important for effective bioavailability enhancement. The enzyme-mediated precipitation of parent drugs from their phosphate prodrugs has been studied and it was concluded that the extent to which a parent drug precipitates during conversion from the prodrug is dependent upon the prodrug to parent absorption rate and also potential precipitation or solubilisation effect of prodrug on parent [14]. For fosamprenavir, the generation of a supersaturated state following conversion has been demonstrated in vitro [15] and this resulted in enhanced absorptive flux across the intestinal mucosa in comparison to non-supersaturated solutions.

**Figure 2:**
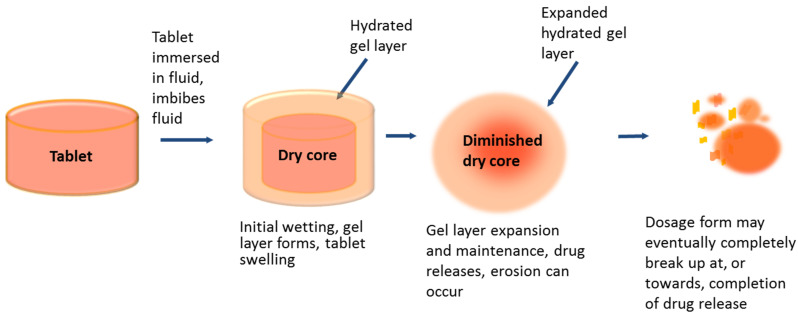
Progressive hydration of a hydrophilic matrix tablet through the process of drug release.

For this prodrug/parent pair, the insoluble parent drug was also shown to have a very short apparent elimination half-life, therefore formulation into an extended release (ER) hydroxypropyl methylcellulose (HPMC) matrix tablet was selected in order to blunt maximum attained plasma concentration (C_max_) and maintain an effective minimum plasma concentration prior to next dose (C_min_) thus ensuring any plasma peak concentration adverse effects are minimized and viral inhibitory levels are sustained between doses [16]. Hydrophilic matrix tablets for oral use containing HPMC as the swelling polymer are commonly used to provide extended drug release through surface diffusion and barrier erosion of the ‘gel layer’. They are designed to swell from the outer surface by polymer hydration and chain relaxation on swallowing, or on contact with a dissolution medium, forming a hydrated gel layer around a dry core (Figure 2) [17]. The gel layer changes over time with the relative position of the erosion and swelling fronts; the dry core becomes depleted over time as polymer at the swelling front becomes hydrated and drug dissolves into the gel [18]. The polymer chains here are strongly entangled but as the gel approaches the erosion front, it becomes more hydrated as the chains disentangle and the polymer dissolves. Drug release is dependent on both the extent of tablet swelling (fluid ingress) and erosion of the gel layer at the tablet’s surface [19,20], and is also affected by the rate of the drug’s diffusion through the hydrated matrix. The solubility of the drug in an aqueous environment will determine the rate at which a drug will diffuse out of a gel matrix; the rate being greater for a soluble compound compared to an insoluble compound, hence the soluble prodrug fostemsavir has a much faster diffusion rate than the insoluble parent.

An ER tablet containing a phosphate ester prodrug carries a risk in that the success of this approach is dependent upon the level of alkaline phosphatase (ALP) expression in the lower GI tract and the ability of the ER tablet to deliver the prodrug at the appropriate rate to the site for conversion to the parent. In addition to being located at the gut wall, alkaline phosphatase is also known to be secreted in bile, and by enterocytes into duodenal fluid, often as complexed forms which are subsequently converted to free enzymes by phospholipase D [21,22].

Activity of ALP in human intestinal fluid aspirates has been demonstrated [14] and it is also detected in the lumenal contents of the large intestine [23]. This availability of ALP in the luminal contents generates an additional risk to the ER delivery of prodrugs, due to the potential for the enzyme to become incorporated within the hydrating gel layer and to promote the hydrolysis of the prodrug to the parent within the gel layer prior to release from the matrix for absorption. If this occurred, the presence of high levels of poorly soluble parent drug in the gel layer, relative to levels of the much more soluble prodrug, would lead to a lower driving force for diffusional release and an altered release profile. Therefore, significant hydrolysis of fostemsavir within the hydrated dosage form can lead to reduced and variable bioavailability. The objective of this work was to investigate the potential for alkaline phosphatase to enter into a hydrating HPMC ER tablet containing fostemsavir and to prematurely convert the prodrug into the parent within the gel layer prior to release of drug from the dosage form.

## 2. Materials and Methods

### 2.1. Materials

Alkaline phosphatase from porcine intestinal mucosa (1.1 units/mg) (ALP), 5-bromo-4-chloro-3-indoyl disodium (BCIP) and nitrotetrazolium blue chloride (NBT) were reagent grade materials from Sigma-Aldrich Chemicals. Hydroxypropyl methylcelluose 2208 (HPMC, Methocel K4M, Colorcon), tromethamine (Tris) (Emprove^®^, Merck, (Darmstadt, Germany) and fosphenytoin sodium (Sigma-Aldrich, Gillingham, UK) were pharmacopoeial grade materials. Universal, full range indicator solutions and potassium dihydrogen orthophosphate dihydrate (analytical grade materials) were purchased from Fisher Scientific UK. Fostemsavir (as tromethamine salt), its parent compound temsavir and metformin hydrochloride were provided by Bristol Myers Squibb (New Brunswick, NJ, USA). Throughout this work, wherever fostemsavir is described it was used as its tromethamine salt but amounts in dosage forms indicated are expressed as free acid.

#### 2.1.1. Preparation of Drug Solutions

Solutions containing either 0.6 mg/mL or 60 mg/mL fostemsavir in pH 7.0 0.05 M tromethamine buffer and 0.6 mg/mL fostemsavir in pH 6.8 0.05 M phosphate buffer were prepared immediately before use, final pH checked and then analysed by HPLC.

#### 2.1.2. Preparation of ER Matrix Tablets

Hydrophilic matrix ER tablets contained fostemsavir (600 mg as free acid), HPMC 2208, microcrystalline cellulose, silicon dioxide and magnesium stearate [24] and were prepared via a dry granulation roller compaction process. Drug and excipients were mixed in a V-cone blender (MB100 with 50 L vessel, Pharmatech, Coleshill, UK) in two steps over 30 min at 20 rpm. A premix of drug, silicon dioxide and magnesium stearate was prepared first then this was combined with the microcrystalline cellulose and the HPMC 2208. This blend was then roller compacted on a Freund Mini or a Freund Labo compactor (SLP, High Wycombe, UK) with a compaction pressure of 5–12 MPa, roll speed 3–5 rpm and a screw feed of 20–30 rpm, producing ribbons of approximately 1.2 mm thickness. Ribbons were milled in a conical sieve mill (Frewitt, Fribourg, Switzerland or SLP, High Wycombe, UK) with a 1.0 mm screen. The resultant granules were lubricated with magnesium stearate in the V-cone blender and compressed into tablets using capsule-shaped tooling (19.0 mm by 9.25 mm) on a single station Manesty ‘F’ tablet press (Manesty, Liverpool, UK) to achieve a tablet hardness of 140–170 N. In the case of alkalinised tablets, to keep tablet surface area to volume ratio constant relative to non-alkalinized tablets, as this is critical parameter for the release rate of drugs from hydrophilic matrix tablets [25], a portion of the fostemsavir was replaced with an equivalent weight of tromethamine (12% *w/w*), but other ingredients remained the same. Although the commercial product is intended to be coated with a non-functional film coat for aesthetic and identification purposes, uncoated tablets were employed in this work to simplify processing of materials for investigative purposes. The film coat on the intended commercial product hydrates and dissolves away quickly, in just a couple of minutes, and has no effect on drug release kinetics (data not shown).

#### 2.1.3. Preparation of Compacts for Substrate Experiments

Compacts (100–200 mg, flat-faced, round, 7–13 mm diameter) based on a blend of HPMC 2208, microcrystalline cellulose, silicon dioxide and magnesium stearate were prepared using a hydraulic press (Graseby Specac, Orpington, UK) to simulate the hydrophilic matrix tablet formulation. Compacts had 0.1% *w*/*w* BCIP and 0.2% *w/w* NBT incorporated for alkaline phosphatase visualization, this ratio selected based on ratios used in Western blot methodology [26]. Fostemsavir was included at 0%, 5.3%, 26.5%, 39.5% and 53.3% *w/w*, with a constant mass of highly water-soluble component in the overall matrix formulation being maintained by adding mannitol to yield a total weight of drug plus mannitol equivalent to 53% *w/w* of drug alone. To explore the effect of microenvironmental pH on ALP activity in the hydrated matrix, 12% *w/w* tromethamine was added to compacts containing 53% *w/w* BMS-A.

To explore whether prodrug parent molecule interfered with ALP activity in the hydrated matrix, 43% *w*/*w* of the parent compound temsavir was added to compacts replacing fostemsavir. Inorganic phosphate ions are known to be able to inhibit the activity of alkaline phosphatase [27]. Hence, to determine if free phosphate, liberated by hydrolysis of the prodrug within the hydrated matrix, was inhibitory to ALP activity in the hydrated compact, potassium dihydrogen phosphate (in an amount equivalent to phosphate liberated by complete hydrolysis of prodrug at 53% *w/w* loading) was included in the matrix instead of fostemsavir.

To investigate the effect of highly water-soluble drug osmolality on ALP activity in the hydrated tablet matrix compacts were prepared substituting metformin hydrochloride for fostemsavir. Its aqueous solubility is similar and has osmolality greater than that of fostemsavir (data not shown).

To determine if a phosphate group attached to a drug molecule other than fostemsavir could affect ALP activity in the hydrated tablet matrix, compacts were prepared substituting a different highly water-soluble phosphate ester prodrug, fosphenytoin sodium [28] (Figure 1), for fostemsavir.

### 2.2. Methods

#### 2.2.1. Drug Release Testing

In vitro dissolution testing of formulations in the absence of ALP was performed at 37 °C using 1000 mL pH 7.0 tromethamine buffer in USP type 1 apparatus at 100 rpm. Quantitation of the amount of dissolved fostemsavir was performed by UV spectrophotometry (λmax 304 nm), employing the linear calibration curve constructed using fostemsavir dissolved in pH 7.0 tromethamine buffer over the range equivalent to 5–120% of amount of drug in the evaluated tablets and compacts. Samples were filtered through 45µm in-line filters prior to analysis.

#### 2.2.2. Determination of Extent of Prodrug Hydrolysis within Hydrated Tablet Gel Layer

Experiments were conducted in duplicate in USP type 1 apparatus employing 450 mL pH 7.0 tromethamine buffer containing 1 mg/mL ALP, maintained at 37 °C. At 3 h, 7 h and 24 h time-points, the basket containing the hydrated tablet were removed from the dissolution apparatus. The tablet, including its gel layer was subjected to homogenisation using a stand-alone disperser (Polytron PT3100) in 500 mL 50:50 methanol: water and a filtered aliquot of this solution was diluted 1 in 5 with the same diluent. At the same time a sample of the dissolution medium was taken, filtered through GHP Acrodisc 0.45 µm membrane syringe filters (Pall/Life Sciences) and diluted 1 in 5 in a 50:50 methanol: water diluent. The 50:50 methanol: water diluent was selected due to its ability to dissolved both fostemsavir and its parent temsavir and for the inhibitory action of organic solvent upon enzyme activity. Both the homogenised tablets and dissolution media samples were analysed by HPLC.

#### 2.2.3. HPLC Analysis of Fostemsavir and Temsavir

HPLC was performed using a C_18_ column (XTerra RP_18_, 150 × 3.9 mm, 5 µm) maintained at ambient temperature employing a variable wavelength detector (G1314A, Agilent) with a detection wavelength of 245 nm. Mobile Phase A, 25 mM ammonium acetate (pH 8.2): methanol (95:5 *v/v*) and Mobile Phase B, methanol, were delivered (Agilent G1310A Quaternary pump) at 0.75 mL/min, according to the following gradient profile: Mobile Phase A and Mobile Phase B solutions initially at 80% and 20%, respectively, proceeded to 0% and 100%, respectively, in a linear profile over 17 min.

#### 2.2.4. Determination of Gel Layer pH Using pH Indicator

Fostemsavir tablets were hydrated in pH 7.0 tromethamine buffer + 0.5% universal indicator at 37 °C, and placebo compacts were hydrated at 37 °C in pH 7.0 tromethamine buffer + 2.0% full range indicator using USP-1 dissolution apparatus. The dosage form was removed from the dissolution media at t = 3, 7 and 24 h. The hydrated gel was photographed, and the colour obtained was compared to a reference chart provided with the full range indicator.

#### 2.2.5. HPMC Compact Enzyme Substrate Experiments

For investigations of mechanism of inhibition of enzyme activity in the hydrated tablet matrix, compacts were hydrated in excess pH 7.0 tromethamine buffer, either with or without ALP at a concentration of 1 mg/mL. The media containing compacts was maintained at 37 °C using a water bath. The colour of the compact was monitored visually over time and the compacts were photographed at 3 and 7 h.

## 3. Results and Discussion

### 3.1. Activity of Alkaline Phosphatase within the Gel Layer of Fostemsavir ER Tablets

**Figure 3:**
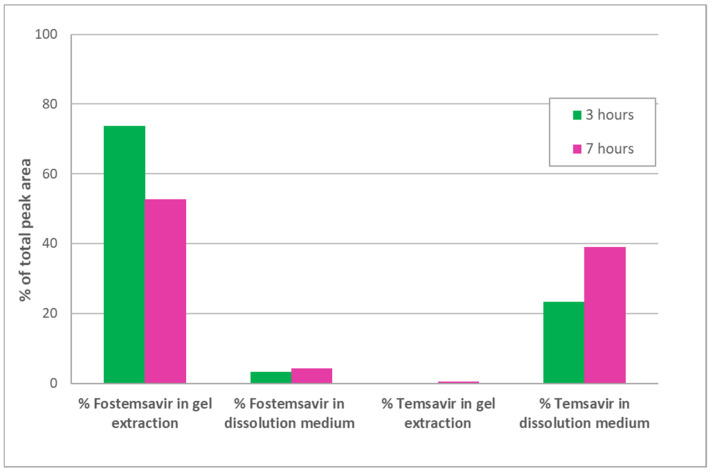
Concentration of fostemsavir and parent temsavir in hydrating ER tablets and dissolution media after 3 h and 7 h hydration.

**Figure 4:**
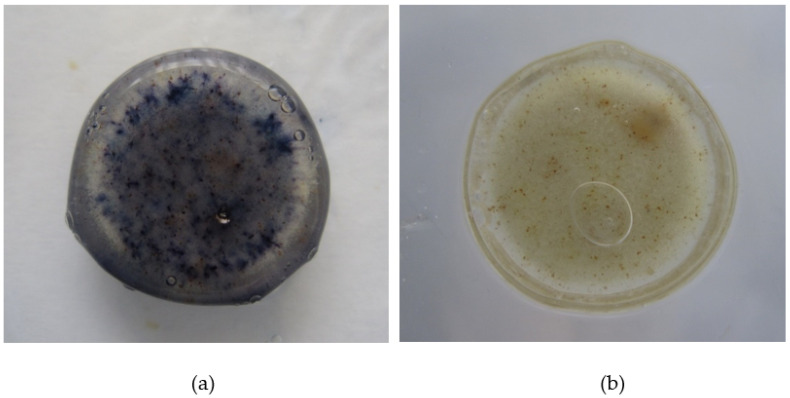
(**a**) Hydrated compact, 1mg/mL, ALP in Tris buffer at 3 h (**b**) Hydrated control, no ALP in Tris buffer at 3 h.

A concern with delivering a phosphate ester prodrug from a hydrophilic matrix tablet is that as the gel layer is formed due to uptake of the aqueous medium in which the dosage form is immersed; if this is intestinal medium in vivo, then ALP present in the gut lumen could be taken up by the dosage form and in situ hydrolysis of the prodrug could occur prematurely before drug is released. When prodrug fostemsavir ER tablets were hydrated in pH 7.0 tromethamine buffer containing ALP at 1 mg/ mL, HPLC analysis revealed less than 2.0% parent temsavir relative to fostemsavir in the tablet as a whole, even after 7 h of hydration, but quantitative conversion for drug released into the dissolution medium (Figure 3). The activity of the enzyme was demonstrated in situ in ER tablets by use of the BCIP/NBT pair originally developed for visualization of ALP in Western blots [26]. 5-bromo-4-chloro-3-indoyl phosphate disodium (BCIP) is a substrate of ALP. The product of this reaction then further reacts to release hydrogen ions which react with nitrotetrazolium blue chloride (NBT) when in solution, forming an insoluble purple product. When drug-free HPMC compacts containing the BCIP/ NBT substrate were hydrated in pH 7.0 tromethamine buffer containing ALP at 1 mg/mL, a purple colouration was observed throughout the gel layer after 3 h, however such colouration was not observed in the control experiment in which HPMC-substrate compacts were hydrated in pH 7.0 tromethamine buffer with no added enzyme (Figure 4). These results confirm that the enzyme present in the hydrating media was incorporated into an HPMC gel layer upon hydration, and that the enzyme was shown to remain active within that environment.

Given that it had been demonstrated that the enzyme was taken up into the gel layer as it evolved, and that the enzyme was active when so incorporated into the gel layer, the low degree of hydrolysis of drug in hydrated tablets was unexpected. Solutions of fostemsavir are weakly acidic and it was considered that this may have an impact on enzyme activity in the gel layer in its presence. Solutions containing 60 mg/mL fostemsavir were found to have a lower rate of conversion to temsavir compared to solutions containing 0.6 mg/mL fostemsavir (Table 1). The corresponding pH values of these solutions were 4.4 and 6.8, respectively, suggesting it may be that environmental pH is a significant factor in inhibiting enzyme activity in the hydrated gel layer where a saturated solution of fostemsavir might be expected to be present.

**Figure 5:**
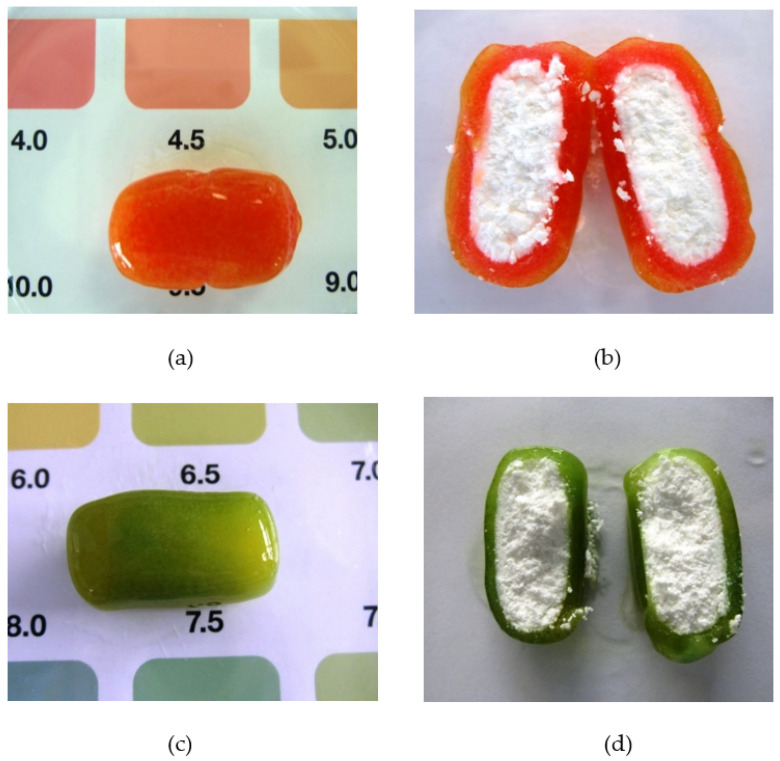
(**a**) Tablet hydrated for 3 h. (**b**) Tablet hydrated for 3 h, pH 4–5 halved to show dry core. (**c**) Tablet hydrated for 3 h. (**d**) Tablet hydrated for 3 h, pH 6.5–7.5, halved to show dry core.

The pH of the gel layer during drug release was demonstrated by hydrating fostemsavir ER tablets in dissolution medium containing universal indicator solution. The pH of the hydrated gel layer was approximately 4–5 through the first 7 h of hydration (Figure 5a,b). The activity of ALP in liberating parent drug from a different phosphate ester prodrug has been shown to decrease with decreasing pH [29] and this could be a reason for the observed decrease in rate of conversion. Hence, the effect of environmental pH within the hydrated gel layer of the tablet on enzymatic drug hydrolysis was further investigated.

### 3.2. Activity of Alkaline Phosphatase within the Gel Layer of Alkalinised Fostemsavir ER Tablets

To investigate whether the inhibition of enzyme activity within the gel layer is caused by simply pH or an additional factor, tromethamine was included in the tablet formulation. Tromethamine has been shown previously to offer a useful buffering option for weak acid drugs in HPMC-based systems. This is because of its good water solubility, its ability to induce a neutral to slightly alkaline buffered microenvironment and its ability to retain hydrated tablet matrix integrity even at high concentrations (up to 50% *w/w*) and to have minimal effect upon gel layer formation [30]. It was confirmed that adding tromethamine to the ER tablet formulation did not change the drug release characteristics, the ability to develop a coherent gel layer and an assessment of gel layer pH by hydration in media containing universal indicator solution showed the inclusion of tromethamine into the tablet matrix at a 12% w/w level within the fostemsavir tablet formulation provided sufficient buffering capacity to maintain the gel layer at pH greater than 6 over 7 h (Figure 5c,d). HPMC compacts containing tromethamine with no fostemsavir present and hydrated in a in pH 7.0 tromethamine buffer containing 1 mg/mL ALP confirmed that a level of 12% w/w tromethamine itself does not affect the level of enzyme activity, as development of a strong purple colouration was observed.

**Figure 6:**
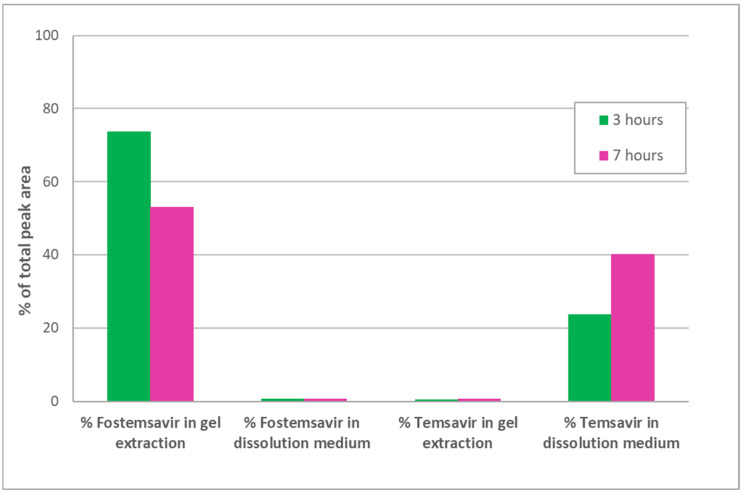
Concentration of prodrug fostemsavir and parent temsavir in fostemsavir + added tromethamine ER tablets, and in dissolution media after 3 h and 7 h hydration.

Extraction and HPLC analysis of fostemsavir and temsavir from the partially hydrated fostemsavir tablets containing the added tromethamine showed a similarly low conversion rate to that seen in non-alkalinised gel layers, with less than 2% conversion at 7 h (Figure 6). This suggests that even if gel layer pH is alkalinised to overcome the known inhibitory effect of acidic pH on enzyme activity, the presence of a saturated solution of fostemsavir in the hydrated gel layer is still exerting an in situ inhibitory effect on the enzyme. Visual observation of compacts containing fostemsavir and pH-adjusted with tromethamine (to assure microenvironmental pH favourable to enzyme activity) along with the BCIP/ NBT probe when hydrated in a pH 7.0 tromethamine buffer with 1 mg/mL ALP exhibited negligible colour development, indicative of very low enzyme activity inside the hydrated matrix.

That this was a consequence of presence of the specific molecule itself and not simply a concentration effect, i.e., a “salting out” of the enzyme, was explored. Compacts containing highly water-soluble metformin hydrochloride as a substitute for fostemsavir, pH-adjusted within the tablet matrix with added tromethamine, were hydrated using pH 7.0 tromethamine buffer in the presence of 1 mg/mL ALP. Metformin hydrochloride was selected for this investigation, as solutions of this highly water-soluble compound have a higher osmolality than fostemsavir (data not shown), providing for a robust assessment of the effect of osmolality.

Visual observation of the compacts revealed the development of a strong purple colouration, indicating that the presence of a highly soluble ingredient with a higher osmolality to fostemsavir appeared not to inhibit enzyme activity, suggesting that the drug does not inhibit ALP in the hydrated tablet gel layer simply by virtue of osmolality effects.

### 3.3. Investigation of the Influence of Phosphate as a Phosphate Prodrug upon Enzyme Activity

**Figure 7:**
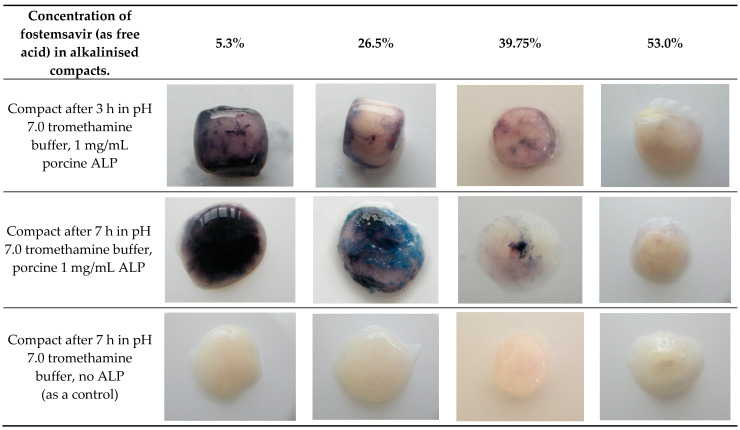
Hydrated compacts containing various concentrations of fostemsavir, pH-adjusted with tromethamine.

When fostemsavir was replaced by an equimolar amount of its parent, then a strong purple colouration developed indicating the presence of the phosphate function on the molecule was important to inhibition of enzyme activity within the hydrated tablet matrix. The inhibitory effect of fostemsavir was further confirmed as demonstrating concentration dependence. When compacts containing various concentrations of fostemsavir and pH-adjusted with tromethamine were hydrated in pH 7.0 tromethamine buffer containing 1 mg/mL ALP, no significant colouration in the compacts containing the highest concentration of fostemsavir was observed, which gradually appeared as a stronger colouration in compacts containing increasingly lower concentrations of the prodrug (Figure 7). The degree of colouration observed between the compacts above shows the amount of fostemsavir present influences the extent of enzyme activity within the gel layer.

As fostemsavir might be considered to essentially be presenting a high level of dissolved phosphate in the gel layer, it could be that simply the high phosphate level in the gel layer might be inhibitory to enzyme activity. To assess this, compacts containing potassium dihydrogen phosphate of a level of phosphate equal to that in a fostemsavir ER matrix tablet formulation were produced. The tablets included tromethamine as a pH adjuster and sufficient metformin hydrochloride to make the compacts the same size as the fostemsavir compacts. These compacts were hydrated in the presence of 1 mg/mL ALP, and visual observation of these hydrated compacts showed a deep purple colouration of the substrate, both at 3 h and 7 h.

This apparent enzyme activity within the gel layer of the compact shows that even though inorganic phosphate has been documented to be inhibitory towards ALP, at the level of phosphate present within a fostemsavir ER tablet formulation, the ALP is still active and there must be another factor having an effect on the ability of fostemsavir itself to inhibit ALP activity in the hydrated tablet matrix.

**Figure 8:**
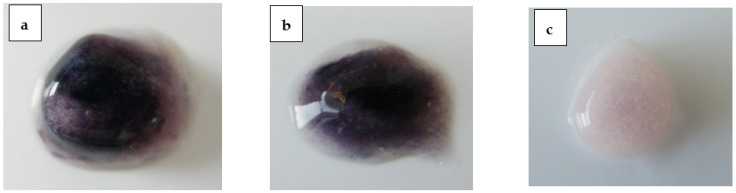
(**a**) Fosphenytoin compact at 3 hours, (**b**) fosphenytoin compact at 7 hours, (**c**) hydrated fosphenytoin compact with no ALP at 7 hours.

Fostemsavir in a hydrated matrix tablets therefore appears itself to be inhibitory to the activity of ALP that enters the matrix tablet with the uptake of the external aqueous medium by the tablet. The effect is demonstrated as not being due to a pH shift within the matrix created by the dissolution of fostemsavir, is not due to increased osmolality within the hydrate matrix due to the prodrug dissolved in the gel layer and is not due to organic phosphate liberated from the prodrug due to its hydrolysis within the matrix. Uniquely the inhibitory action it appears to be a property of the prodrug itself, as another phosphate ester prodrug, fosphenytoin did not show the effect, and increasing levels of fostemsavir within a tablet matrix appeared to increasingly reduce the activity of ALP as demonstrated by the reduced generation of colour from the BCIP/NBT reagent within the matrix (Figure 8). Consequently, it is feasible to deliver this phosphate ester prodrug via a hydrophilic matrix tablet, despite the uptake of ALP into the hydrating matrix.

## 4. Conclusions

This study has confirmed that for hydrophilic matrix tablets, although enzyme activity may occur within the hydrated gel layer, inhibition of ALP activity is occurring within the gel layer of ER hydrophilic matrix tablet formulations containing fostemsavir, and several possible mechanisms have been investigated.

The active ingredient creates an acidic environment in the hydrated tablet gel layer. The low pH in the gel layer alone thus provided may be sufficient to inactivate the enzyme; however, it has been shown that there is also a lack of activity of the enzyme in alkalinised hydrated gels containing the prodrug. Potential properties which may be implicated include high solubility of API leading to a high osmolality in the gel layer, or a self-inhibitory mechanism in which inorganic phosphate generated through prodrug to parent conversion reaches a level in the gel layer where inhibition of enzyme activity occurs. Both these potential causes of enzyme inhibition within the gel layer were investigated; a similarly highly soluble API, metformin hydrochloride possessing a high osmolality in the gel layer, did not inhibit enzyme action, nor does inorganic phosphate at a level calculated to be available after prodrug to parent conversion.

With a phosphate function attached to the highly water-soluble prodrug, its saturated solution in the hydrated gel layer of the tablet might be associated with the inhibition of ALP in that gel layer. To explore this, a different highly water-soluble phosphate ester prodrug, fosphenytoin, was used in place of fostemsavir in tablet formulations. It was found that this did not inhibit the activity of ALP. The inhibitory activity of fostemsavir towards ALP in the hydrated gel layer of hydrophilic matrix tablet formulations in an apparent concentration dependent manner appears to be a property intrinsic to the fostemsavir molecule and conveniently allows for hydrophilic matrix tablets as a suitable modified release approach for this compound.

Finally, another highly water-soluble organic phosphate-containing drug, fosphenytoin sodium, was used in the formulation, as a surrogate for fostemsavir. Compacts were made containing fosphenytoin sodium to provide the same level of organic phosphate as the 53% w/w fostemsavir formulation, and with tromethamine included as a pH adjuster. These compacts were hydrated in the presence of 1 mg/mL ALP, and visual observation of these hydrated compacts showed a deep purple colouration of the substrate, both at 3 h and 7 h (Figure 8), confirming ALP activity in the hydrated matrix and that the alternate phosphate ester prodrug fosphenytoin sodium dissolved in the hydrated matrix and providing equivalent levels of phosphate activity was not inhibitory towards ALP present in the hydrated matrix.

## Figures and Tables

**Table 1:** Effect of fostemsavir concentration on solution pH and conversion rate of prodrug to parent in pH 7.0 Tris buffer.

Fostemsavir Concentration	60 mg/mL	0.6 mg/mL
pH	4.4	6.8
Estimated conversion rate	0.02%/h	>39%/h

## Data Availability

Data is archived at Bristol Myers Squibb, please contact the authors.
